# Dissecting the function of networks underpinning language repetition

**DOI:** 10.3389/fnhum.2014.00727

**Published:** 2014-10-02

**Authors:** Marcelo L. Berthier, Matthew A. Lambon Ralph

**Affiliations:** ^1^Cognitive Neurology and Aphasia Unit, Centro de Investigaciones Médico-Sanitarias, University of MálagaMálaga, Spain; ^2^Neuroscience and Aphasia Research Unit, School of Psychological Sciences, University of ManchesterUK

**Keywords:** repetition, arcuate fasciculus, dorsal stream, ventral stream, language, aphasia, conduction aphasia, temporal lobe

In the nineteenth century, ground-breaking observations on aphasia by Broca (1865) and Wernicke (1906) suggested that language function depends on the activity of the cerebral cortex. At the same time, Wernicke (1906) and Lichtheim (1885) also elaborated the first large-scale network model of language which incorporated long-range and short-range (transcortical connections) white matter pathways in language processing. The arcuate fasciculus (dorsal stream) was traditionally viewed as the major language pathway for repetition, but scientists also envisioned that white matter tracts traveling through the insular cortex (ventral stream) and transcortical connections may take part in language processing. Modern cognitive neuroscience has provided tools, including neuroimaging, which allow the *in vivo* examination of short- and long-distance white matter pathways binding cortical areas essential for verbal repetition. However, this state of the art on the neural correlates of language repetition has revealed contradictory findings, with some researchers defending the role of the dorsal and ventral streams, whereas others argue that only cortical hubs (Sylvian parieto-temporal cortex [Spt]) are crucially relevant.

An integrative approach would conceive that the interaction between these structures is essential for verbal repetition. For instance, different sectors of the cerebral cortex (e.g., Spt, inferior frontal gyrus/anterior insula) act as hubs dedicated to short-term storage of verbal information or articulatory planning and these areas in turn interact through forward and backward white matter projections. Importantly, white matter pathways should not be considered mere cable-like connections as changes in their microstructural properties correlate with focal cortical activity during language processing tasks.

Despite considerable progress, many outstanding questions await response. The articles in this Research Topic tackle many different and critical new questions, including: (1) how white matter pathways instantiate dialogues between different cortical language areas; (2) what are the specific roles of different white matter pathways in language functions in normal and pathological conditions; (3) what are the language consequences of discrete damage to branches of the dorsal and ventral streams; (4) what are the consequences (e.g., release from inhibition) of damage to the left white matter pathways in contralateral ones and viceversa; (5) how these pathways are reorganized after brain injury; (5) can the involvement/sparing of white matter pathways be used in outcome prediction and treatment response; and (5) can the microstructure of white matter pathways be remodeled with intensive rehabilitation training or biological approaches.

This Research Topic includes original studies, and opinion and review articles which describe new data as well as provocative and insightful interpretations of the recent literature on the role of white matter pathways in verbal repetition in normal and pathological conditions. A brief highlight summary of each is provided below.

## Articles

Opinion Article, Published on 29 Jul 2013

**The anatomo-functional connectivity of word repetition: insights provided by awake brain tumor surgery**

Sylvie Moritz-Gasser and Hugues Duffau

In this opinion article, Moritz-Gasser and Duffau (2013) provide important insights on the role of the strong interaction of dorsal and ventral streams in word repetition. The authors discuss their pioneering results obtained through direct electrical stimulation of white matter pathways in awake patients during brain tumor surgery.

Review Article, Published on 12 Jul 2013

**Language repetition and short-term memory: an integrative framework**

Steve Majerus

Short-term maintenance of verbal information is crucially important for efficient language repetition of complex information. In a comprehensive review, Majerus (2013) presents an integrative framework aimed at bridging research in the language processing and short-term memory fields.

Hypothesis and Theory Article, Published on 02 Oct 2013

**Mapping a lateralization gradient within the ventral stream for auditory speech perception**

Karsten Specht

Specht (2013) analyses the results from several complementary functional neuroimaging studies with the aim to trace the hierarchical processing network for speech comprehension within the left and right hemisphere. The author pays particular attention to role of the temporal lobe and the ventral stream in auditory speech perception.

Original Research Article, Published on 05 Sep 2013

**Articulation-based sound perception in verbal repetition: a functional NIRS study**

Sejin Yoo and Kyoung-Min Lee

Using functional near-infrared spectroscopy, Yoo and Lee (2013) examine healthy subjects while repeating pseudowords and words. This study reveals that passive listening without repetition to various sounds (natural environmental sounds, animal vocalizations, and human non-speech sounds) as well as articulation activate neural circuits that include both inferior frontal regions.

Original Research Article, Published on 26 Aug 2013

**The roles of the “ventral” semantic and “dorsal” pathways in conduite d'approche: a neuroanatomically-constrained computational modeling investigation**

Taiji Ueno and Matthew A. Lambon Ralph

In a computational modeling investigation of the dual dorsal-ventral pathway implicated in verbal repetition, Ueno and Lambon Ralph (2013) demonstrate that the successful phonetic approximations to target words (*conduite d'approche*), typically observed in patients with conduction aphasia and damage to the dorsal pathway (arcuate fasciculus), relies on the complementary activity of the ventral semantic stream.

Original Research Article, Published on 18 Oct 2013

**Repeating with the right hemisphere: reduced interactions between phonological and lexical-semantic systems in crossed aphasia?**

Irene De-Torres, Guadalupe Dávila, Marcelo L. Berthier, Seán Froudist Walsh, Ignacio Moreno-Torres and Rafael Ruiz-Cruces

In this study, De-Torres et al. (2013) show that repetition after subcortical lesions involving the dorsal and ventral streams in patients who are right-hemisphere dominant for language is not heavily influenced by lexical-semantic variables as is regularly reported in similar cases with left hemisphere damage.

Original Research Article, Published on 10 Dec 2013

**Predicting speech fluency and naming abilities in aphasic patients**

Jasmine Wang, Sarah Marchina, Andrea C. Norton, Catherine Y. Wan and Gottfried Schlaug

The identification of reliable biomarkers that predict the degree of chronic speech fluency/language impairment and potential for improvement after stroke is paramount. In this study, Wang et al. (2013) demonstrate that lesion load in the arcuate fasciculus (dorsal stream) is the best anatomical marker at stratifying patients into different outcome groups with high accuracy for speech fluency and naming.

Original Research Article, Published on 31 Jan 2014

**Sensory-to-motor integration during auditory repetition: a combined fMRI and lesion study**

‘Ōiwi Parker Jones, Susan Prejawa, Tom Hope, Marion Oberhuber, Mohamed L. Seghier, Alex P. Leff, David W. Green and Cathy J. Price

On examining sensory-to-motor integration during auditory repetition in healthy subjects and aphasic patients, Parker Jones et al. (2014) find that normal and abnormal repetition of pseudowords correlate with activity of the arcuate fasciculus, but is unrelated to the activity of different cortical areas.

Original Research Article, Published on 19 Dec 2013

**Dissociated repetition deficits in aphasia can reflect flexible interactions between left dorsal and ventral streams and gender-dimorphic architecture of the right dorsal stream**

Marcelo L. Berthier, Seán Froudist Walsh, Guadalupe Dávila, Alejandro Nabrozidis, Rocio Juarez y Ruiz de Mier, Antonio Gutiérrez, Irene De Torres, Francisco Alfaro, Natalia García-Casares and Rafael Ruiz-Cruces

Using multimodal neuroimaging, Berthier et al. (2013) evaluate the neural correlates of repetition performance in two aphasic patients matched for lesion volume (a female patient with preserved repetition and a male patient with impaired repetition). Dissociated repetition deficits in these cases are probably reliant on flexible interactions between left dorsal stream and left ventral stream and on gender-dimorphic architecture of the right dorsal stream.

Original Research Article

**Dissecting the functional anatomy of auditory word repetition**

Thomas Matthew Hadley Hope, Susan Prejawa, ‘Ôiwi Parker Jones, Marion Oberhuber, Mohamed L. Seghier, David W. Green and Cathy J. Price

Hope et al. (2013) use a single, multi-factorial, within-subjects fMRI design to identify those regions, and to functionally distinguish the multiple linguistic and non-linguistic processing areas, that are all involved in repeating back heard words. They find that repetition activates components of regions not hitherto implicated in word repetition. Thus, these novel findings challenge some of the commonly held opinions on the functional anatomy of language.

### Conflict of interest statement

The authors declare that the research was conducted in the absence of any commercial or financial relationships that could be construed as a potential conflict of interest.

## References

[B1] Berthier M. L. Walsh S. F. Dávila G. Nabrozidis A. de Mier R. J. R. Gutiérrez A. (2013). Dissociated repetition deficits in aphasia can reflect flexible interactions between left dorsal and ventral streams and gender-dimorphic architecture of the right dorsal stream. Front. Hum. Neurosci. 7: 873 10.3389/fnhum.2013.00873 24391569 PMC3867969

[B1a] Broca P. (1865). Sur la faculté du langage articulé. Bull. Soc. Anthrop. (Paris) 6, 337–393

[B2] De-Torres I. Dávila G. Berthier M. L. Walsh S. F. Moreno-Torres I. Ruiz-Cruces R. (2013). Repeating with the right hemisphere: reduced interactions between phonological and lexical-semantic systems in crossed aphasia? Front. Hum. Neurosci. 7:675 10.3389/fnhum.2013.00675 24151460 PMC3798981

[B3] Hope T. M. H. Prejawa S. Parker Jones O. Oberhuber M. Seghier M. L. Green D. W. (2013). Dissecting the functional anatomy of auditory word repetition. Front. Hum. Neurosci. 8:246 10.3389/fnhum.2014.00246 24834043 PMC4018561

[B5a] Lichtheim L. (1885). On aphasia. Brain 7, 433–484

[B5] Majerus S. (2013). Language repetition and short-term memory: an integrative framework. Front. Hum. Neurosci. 7:357 10.3389/fnhum.2013.00357 23874280 PMC3709421

[B6] Moritz-Gasser S. Duffau H. (2013). The anatomo-functional connectivity of word repetition: insights provided by awake brain tumor surgery. Front. Hum. Neurosci. 7:405 10.3389/fnhum.2013.00405 23908617 PMC3725408

[B7] Parker Jones O. Prejawa S. Hope T. Oberhuber M. Seghier M. L. Leff A. P. (2014). Sensory-to-motor integration during auditory repetition: a combined fMRI and lesion study. Front. Hum. Neurosci. 8:24 10.3389/fnhum.2014.00024 24550807 PMC3908611

[B8] Specht K. (2013). Mapping a lateralisation gradient within the ventral stream for auditory speech perception. Front. Hum. Neurosci. 7:629 10.3389/fnhum.2013.00629 24106470 PMC3788379

[B4] Ueno T. Lambon Ralph M. A. (2013). The roles of the “ventral” semantic and “dorsal” pathways in conduite d'approche: a neuroanatomically-constrained computational modeling investigation. Front. Hum. Neurosci. 7:422 10.3389/fnhum.2013.00422 23986670 PMC3752442

[B9] Wang J. Marchina S. Norton A. C. Wan C. Y. Schlaug G. (2013). Predicting speech fluency and naming abilities in aphasic patients. Front. Hum. Neurosci. 7:831 10.3389/fnhum.2013.00831 24339811 PMC3857577

[B9a] Wernicke C. (1906). Der aphasische Symptomencomplex, in Die duetsche Klinik am Eingange des 20: Jahrhunderts, Vol. 6, eds von Leyden E. Klemperer F. (Berlin: Urban and Schwarzenberg), 487–566

[B10] Yoo S. Lee K.-M. (2013). Articulation-based sound perception in verbal repetition: a functional NIRS study. Front. Hum. Neurosci. 7:540 10.3389/fnhum.2013.00540 24046741 PMC3763229

